# Current trends in theranostic applications of extracellular vesicles in cancer

**DOI:** 10.3389/fonc.2025.1592006

**Published:** 2025-06-03

**Authors:** Yazan Almasry, Fayrouz Mustafa, Mohammed Alfuwais, Sara AlNachef, Hager Mohamed, Nusaibah S. Gaber, Mohammed Imran Khan, Islam M. Saadeldin, Ahmed Yaqinuddin

**Affiliations:** ^1^ College of Medicine, Alfaisal University, Riyadh, Saudi Arabia; ^2^ Research Center, King Faisal Specialist Hospital and Research Centre, Jeddah, Saudi Arabia; ^3^ Comparative Medicine Department, King Faisal Specialist Hospital and Research Centre, Riyadh, Saudi Arabia

**Keywords:** extracellular vesicles, cancer therapy, biomarkers, exosomes, tumor microenvironment, precision medicine

## Abstract

**Background:**

Extracellular vesicles (EVs) play an integral role in cancer biology, influencing tumor progression, metastasis, and tumor microenvironment. Due to their distinctive molecular composition, including proteins, nucleic acids, and lipids, EVs present a promising candidate for cancer diagnostics and precision therapeutics.

**Methods:**

This review was conducted by looking up recent studies obtained through PubMed, Scopus, and Web of Science databases using targeted keywords such as “Extracellular Vesicles,” “Cancer Therapy,” “Biomarkers,” “Exosomes,” “Tumor Microenvironment,” and “Precision Medicine.” From an initial 4,320 articles identified, 427 were screened after applying publication filters, resulting in the inclusion of 298 articles relevant to EV isolation, characterization, diagnostic sensitivity, specificity, and therapeutic efficacy.

**Results:**

Biomarkers derived from EVs derived across various cancers showed high diagnostic performance. For example, four miRNA EVs showing sensitivity and specificity of 98% and 96% respectively was found in breast cancer. EV-RNA and surface antigen analyses for hepatocellular carcinoma with 93.8% sensitivity and 74.5% specificity. Additionally, EV biomarker cancers of the colorectal microRNA miR-23a and miR-301a had 89% sensitivity and >70% specificity. EVs in a therapeutic context were an effective drug delivery system for enhancing precision of chemotherapy and immunotherapy with reduced systemic toxicity.

**Conclusion:**

The theranostics of EVs provide great capacity for early cancer diagnosis and personalized treatment based on their high diagnostic sensitivity and specificity. Future standardization protocols are essential to translate EV technologies into clinical oncology.

## 1 Introduction

Cancer is currently the fifth leading cause of mortality (1). This onus is predicted to increase to 35 million cases by 2050, due to reasons such as aging, population growth, and increasing prevalence of risk factors like obesity, tobacco use, and air pollution (1, 2). Despite ameliorations in cancer therapy, critical challenges prevail in financing its management, with only 39% of countries including basic cancer treatment in healthcare coverage and even fewer providing palliative care. This disparity is echoed further in low-income countries, where late diagnoses and limited access to treatment lead to higher mortality rates (1, 3). Extracellular vesicles (EVs), lipid-bilayer structures secreted by most cells, have recently gained attention for their potential in cancer diagnosis and therapy. These vesicles, ranging from 50 nm to 10 µm in size, are categorized into exosomes, microvesicles (MVs), and apoptotic bodies (ApoBDs) based on their size, biogenesis, and contents (4–6). Their ability to carry diverse cargo, including genetic, protein, and lipid material, makes them versatile tools for intercellular communication and disease spread (7, 8). Exosomes, the smallest subtype, are notable for their ability to cross the blood-brain barrier, enabling targeted drug delivery for neurological diseases and inflammation (5, 6). Likewise, MVs and ApoBDs play important roles in cellular communication and tumor progression, though further research is needed to fully explicate their roles (9–11). In the context of cancer, EVs have been shown to have a significant function in tumor progression and communication within the tumor microenvironment (TME). Cancer-derived EVs carry a diverse number of nucleic acids, proteins, and lipids, such as microRNAs, mutated epidermal growth factor receptors (EGFR), and vascular endothelial growth factors (VEGF), that facilitate intercellular signaling, immune modulation, and the advancement of aggressive phenotypes (12–14). Furthermore, EVs contribute to the horizontal transfer of oncogenic traits and mitigate processes like epithelial-mesenchymal transition (EMT), exacerbating cancer cell invasiveness and metastasis (15). These special characteristics make EVs central components of both cancer pathogenesis and targets for therapeutic intervention. The aim of this article is to provide a comprehensive review of the most recent data regarding the use of EVs in the diagnostic and therapeutic aspects of cancer as well as their clinical applications.

## 2 Methodology

This literature review of current trends in the theranostic applications of EVs in cancer, using articles from PubMed, Scopus, and Web of Science databases. Using keywords including “Extracellular Vesicles,” “Cancer Therapy,” “Biomarkers,” “Exosomes,” “Tumor Microenvironment,” and “Precision Medicine,” yielded approximately 4,320 articles. After applying filters to retain only English-language, systematic reviews, meta-analyses, reviews, and randomized clinical trials published in the last ten years, the number of articles was narrowed down to 427. Following an independent review of the titles, abstracts, and full texts for relevance and alignment with the review’s objective, 298 articles were ultimately included as references in this paper. Primary areas examined included methods of EV isolation and characterization, diagnostic potential (with emphasis on sensitivity and specificity), therapeutic utility of EV-based approaches, and advancements in digital imaging and AI-supported techniques for EV analysis. The table highlighted in future perspectives was aimed at analyzing clinical trials on the clinical applications of extracellular vesicles in cancer. The table was done based on active trials found on clinicaltrials.gov as of January 14th, 2025. The search included keywords like “exosomes,” extracellular vesicles,” and “cancer.” The search excluded studies not related to cancer, and non-diagnostic or therapeutic applications, yielding a total of 58 clinical trials. The table was made to identify the cancer type and subtype, the clinical use including a description of the use, and trial status with NCT identifiers.

## 3 Biogenesis of extracellular vesicles

**Figure 1:**
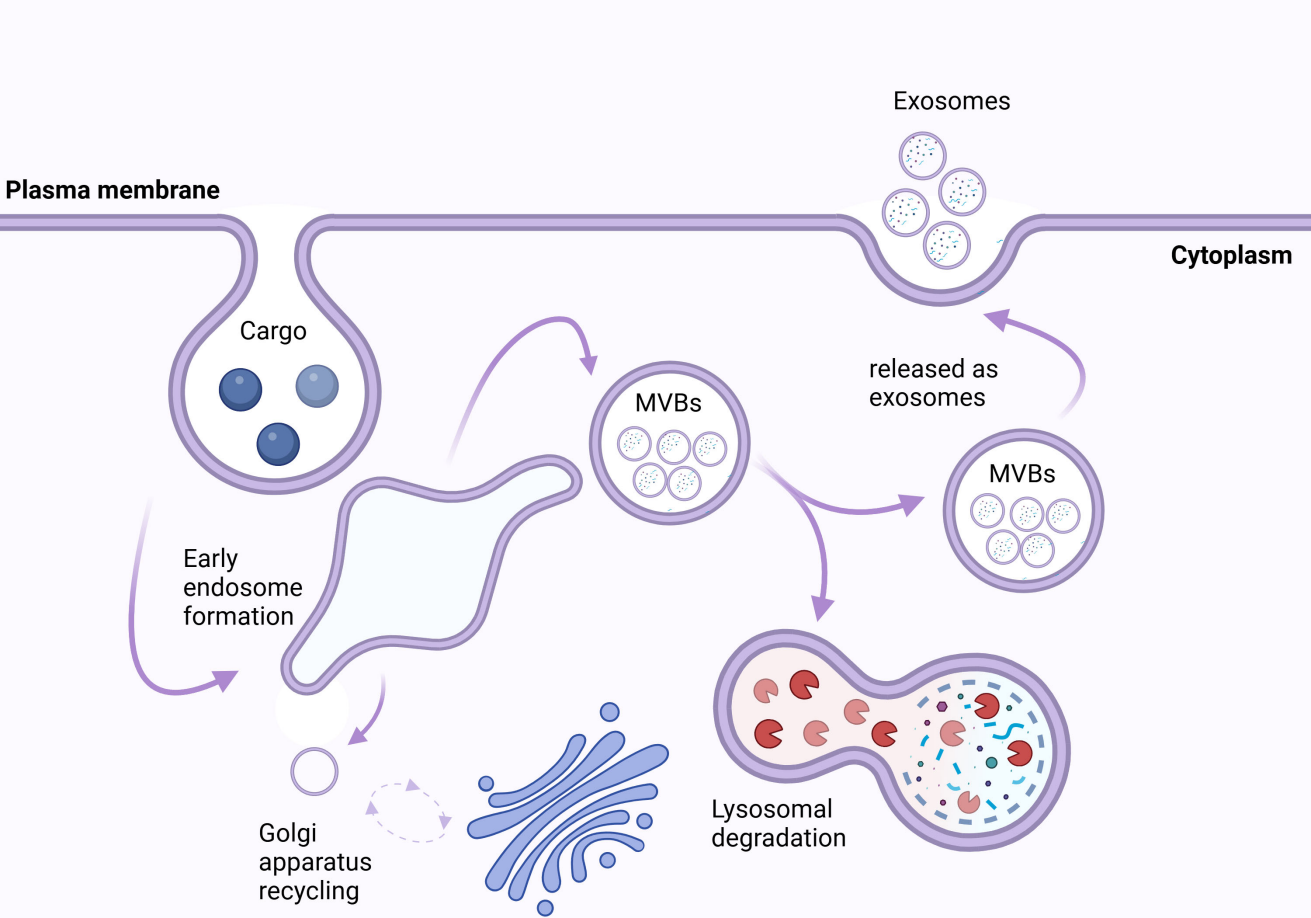
Biogenesis and Fate of Exosomes. The intracellular trafficking and fate of exosomes begins at the plasma membrane, where cargo (e.g., proteins, lipids, nucleic acids) is internalized via endocytosis, forming early endosomes. These early endosomes mature into multivesicular bodies (MVBs) containing intraluminal vesicles. MVBs have two potential fates: they can fuse with lysosomes, leading to lysosomal degradation, or they can fuse with the plasma membrane, resulting in the release of exosomes into the extracellular space. Additionally, vesicular trafficking is regulated by the Golgi apparatus, which recycles membrane components to maintain cellular homeostasis. Exosomes serve as critical mediators of cell-cell communication by transferring their cargo to recipient cells (20).

EVs are classified into a variety of subtypes based on their size, origin, and function. These subtypes include microvesicles (MVs) (0.1–0.35 µm), apoptotic bodies (0.8–5 µm), and small EVs (50–120 nm) (16, 17). Whereas MVs are the result of the plasma membrane’s outward budding (18), apoptotic bodies are formed during programmed cell death (19). In contrast, exosomes are nanoscale vesicles that are produced from multivesicular bodies (MVBs) within late endosomes as shown in **Figure 1** (21).

### 3.1 Exosomes biogenesis

When endocytosis occurs, early endosomes are created that can recycle cargo, break it down via lysosomes, or grow into MVBs with intraluminal vesicles (ILVs) that release exosomes when they fuse with the plasma membrane (**Figure 1**) (22, 23). Endosomal trafficking involves changes in endosomal composition, such as replacing sphingomyelin with ceramide and Rab5 with Rab11, promoting downstream transport and arrangement (22).

#### 3.1.1 ESCRT-dependent pathway

MVB maturation, ILV formation, and cargo recognition are all regulated by the endosomal sorting complex required for transport (ESCRT). ESCRT-0 (Vps27/Hrs.) clusters ubiquitinated cargo, ESCRT-I (Vps23/TSG101) and ESCRT-II (Vps36/EAP45) encourage vesicle budding, and ESCRT-III (SNF7/CHMP4) causes ILV scission, which is completed by ATPase Vps4, whereas (22, 24). Exosome production is enhanced by other regulators, such as the syndecan-syntenin-ALIX pathway, especially in viral pathogenesis and cancer. Epstein-Barr Virus (EBV) uses this mechanism to load latent membrane protein 1 (LMP1) into exosomes, facilitating immune evasion, while oncogenic SRC kinase activates it in breast cancer cell lines like MCF7 cells (25–28). Additionally, Charcot-Marie-Tooth disease type 1C is caused by mutations in the SIMPLE protein that interfere with MVB formation (22, 29).

#### 3.1.2 ESCRT-independent pathway

Other pathways include Rab31, which improves the exosomal packaging of the epidermal growth factor receptor (EGFR) in cancer cells, and Ceramide, which promotes ILV budding (26, 30, 31). Moreover, tetraspanins (CD63, CD81, and CD9) aid in exosome release and cargo sorting, and CD63 controls PMEL loading in melanocytes (26, 32).

### 3.2 Cargo sorting and release

Proteins, lipids, and nucleic acids must all be drawn in via different endosomal sorting processes prior to the production of inward vesicles. For instance, Monoubiquitination is used in cargo sorting to direct proteins into ILVs via ESCRT-0, and deubiquitination takes place prior to exosomal release (22). In melanoma, phosphorylated Vps27/Hrs. promotes the loading of PD-L1 into exosomes, preventing T-cell migration and causing resistance to anti-PD-1 therapy (22, 33). Although the exact mechanisms are still unknown, RNA-binding proteins (RBPs) also mediate RNA inclusion in exosomes (34).

The release of exosomes is dependent on Rab GTPases and SNARE proteins. V-SNAREs (VAMP7, VAMP8) mediate fusion by interacting with t-SNAREs (syntaxins, SNAPs) (22, 34). Hepatitis C transmission via exosomes, for instance, is influenced by syntaxin 4, whereas vesicle docking is controlled by Rab27a/b and Rab35. In cancer and neurodegeneration, exosome secretion is modulated by changes such phosphorylation and O-GlcNAcylation (23). The therapeutic potential of microRNAs (like miR-134 and miR-135b) and long non-coding RNAs (such PVT-1 and HOTAIR) is highlighted by their influence on exosome dynamics (22). These mechanisms highlight the intricate control of exosome release and its relevance in disease contexts.

### 3.3 Exosome cargo uptake

**Figure 2:**
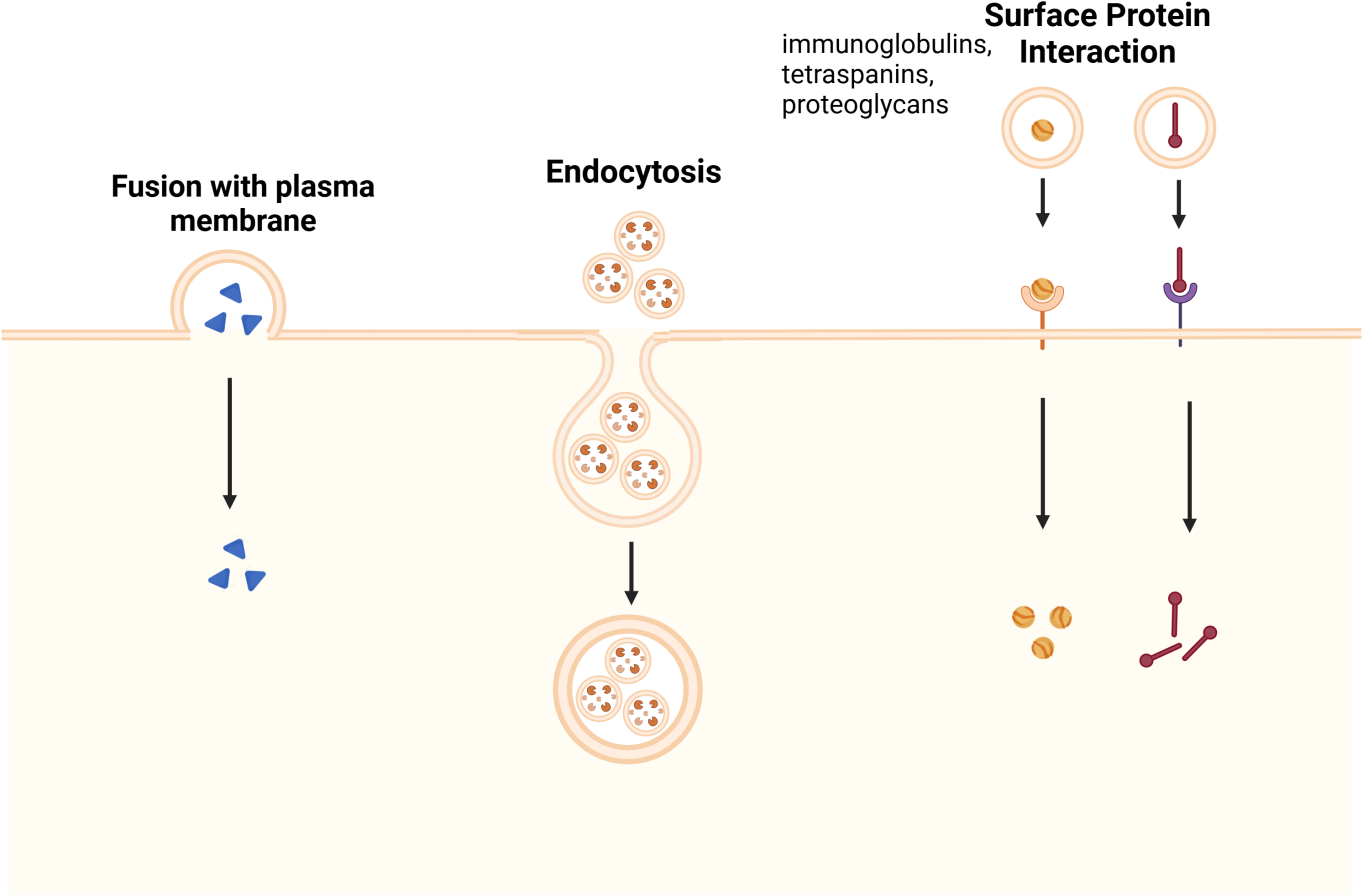
Mechanisms of Exosome Uptake by Recipient Cells. Exosome uptake occurs through three main pathways: fusion with the plasma membrane, where exosomes directly merge with the recipient cell’s membrane, releasing their cargo into the cytoplasm; endocytosis, in which exosomes are internalized via clathrin-mediated endocytosis, macropinocytosis, or caveolin-dependent uptake, leading to intracellular signaling; and surface protein interaction, where exosomal surface proteins, such as immunoglobulins, tetraspanins, and proteoglycans, engage with specific receptors on the recipient cell membrane, triggering signaling cascades or receptor-mediated endocytosis. These uptake mechanisms play essential roles in various physiological and pathological processes, including immune modulation, tissue regeneration, and disease progression (35).

Through membrane fusion or endocytosis, EV cargo directly affects recipient cells, in contrast to receptor-mediated endocytosis. Tetraspanins, proteoglycans, and lectins are examples of surface proteins that help with targeting (**Figure 2**). The therapeutic importance of exosomal ligands PD-L1, TNF, FasL, and TRAIL in cancer comes from their interactions with tumor receptors (22, 26). These findings about the synthesis, sorting, and uptake of exosome emphasize their importance in both health and illness, offering prospects for therapeutic intervention.

## 4 Role of extracellular vesicles in tumor progression and metastasis

Because exosomes are vital for the crosstalk between cells either in the healthy or diseased state, it is crucial to examine their contribution in cancer progression (36). The released exosomes into the extracellular space by exocytosis carry host cell-specific cargo from the host cell that are internalized by recipient cells through endocytosis (37). Within the TME, this promotes interactions between TME cells and cancer stem cells (CSCs) (38). CSCs are subpopulations of cancer cells that have the capacity to self-renew and differentiate into many lineages promoting tumor development and heterogeneity (39). While conventional treatments focus on the tumor’s mass, the resilience of CSCs causes spread and recurrence (36).

Exosomes derived from CSCs can increase tumor aggressiveness by altering the TME, transporting bioactive chemicals to surrounding cells, and promoting tumor development and metastasis (40, 41). Within the TME, HCC-derived exosomes, for example, have been shown to transport tumor-promoting miRNAs like miR-21 and long non-coding RNAs (lncRNAs) like TUC339 that promote cell proliferation while inhibiting tumor-suppressive signaling pathways (36). Additionally, CSC-derived exosomes promote epithelial-mesenchymal transition (EMT) by upregulating TGFβ1, a key regulator of metastasis, and proteases such as matrix metalloproteinase-9 (MMP9) enhancing ECM degradation (36). These mechanisms collectively facilitate tumor cell invasion, dissemination, and the formation of pre-metastatic niches.

EVs released from cancer cells contain heat shock proteins (HSPs) such as HSP70 and HSP90 that maintain protein stability and prevent apoptosis under radiation or chemotherapy-induced damage (41). Therefore, EVs containing HSPs can enhance tumor cell motility, invasiveness, and metastasis, making EV-mediated communication a potential therapeutic strategy to overcome treatment resistance and improve patient outcomes (41).

EVs in the tumor microenvironment are crucial for cancer cell metabolic reprogramming, influencing angiogenesis and immune modulation (40). They also contribute to the remodeling of the extracellular matrix, facilitating cancer cell invasion and dissemination (42). Understanding these interactions can lead to targeted therapies to inhibit tumor progression and metastasis, thereby offering novel strategies for cancer treatment (43).

## 5 Methods of extracellular vesicles concentration and isolation

**Figure 3:**
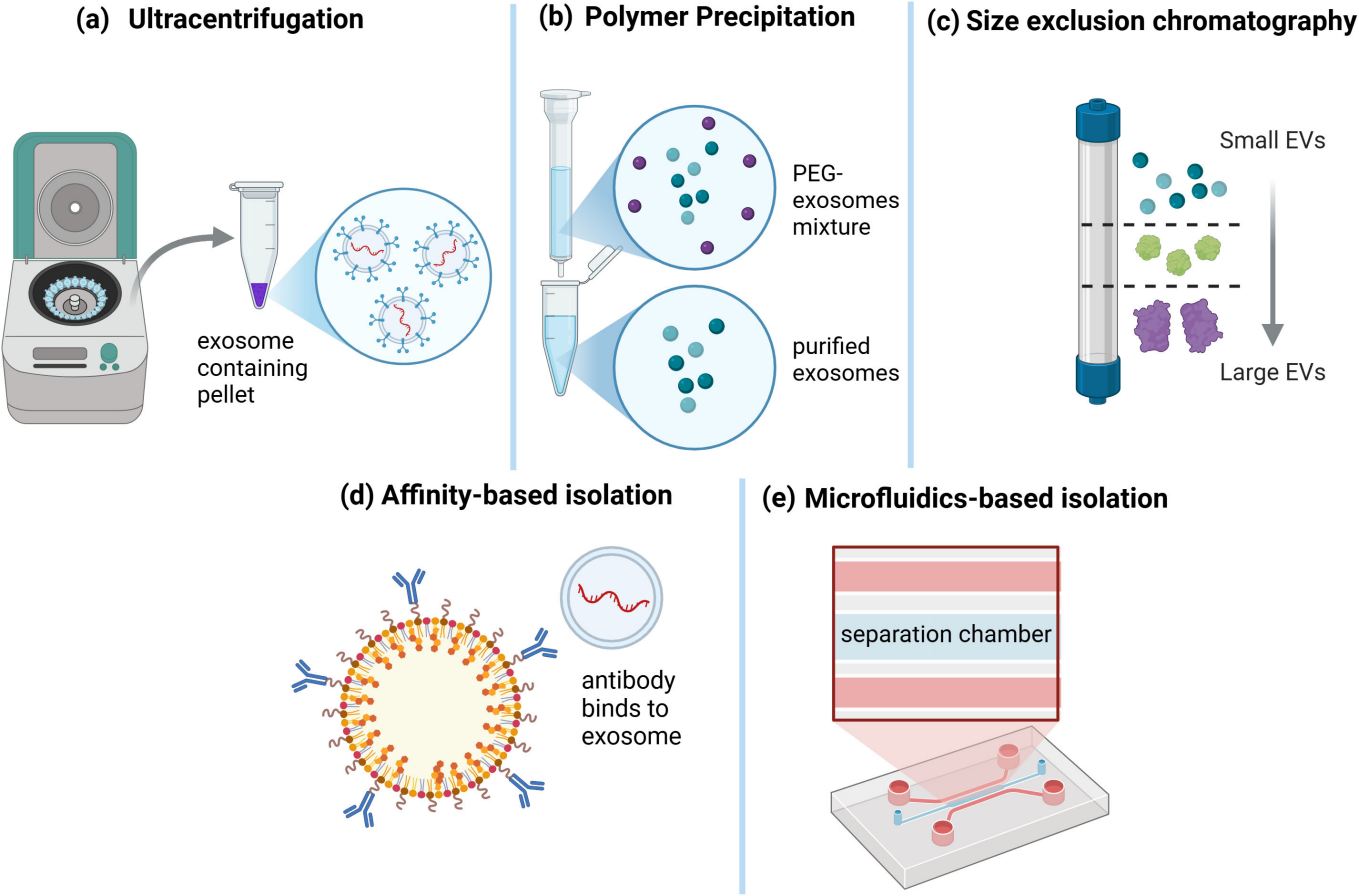
Exosome Isolation Techniques. Various methods have been developed for exosome isolation. **(a)** Ultracentrifugation is a widely used technique that involves high-speed centrifugation to pellet exosomes from biological fluids. **(b)** Polymer precipitation, using agents such as polyethylene glycol (PEG), facilitates exosome aggregation and subsequent precipitation. **(c)** Size exclusion chromatography separates exosomes based on their size, allowing for the enrichment of small extracellular vesicles (EVs) while removing larger particles. **(d)** Affinity-based isolation employs antibodies or ligands that specifically bind to exosomal surface proteins, enabling targeted capture. **(e)** Microfluidics-based isolation utilizes specialized lab-on-a-chip devices with separation chambers designed for high-throughput and precise exosome isolation. These diverse techniques cater to different research and clinical applications, depending on the required purity, yield, and specificity (44).

Several approaches have been developed to efficiently concentrate and isolate EVs from biological fluids by utilizing an EV feature that separates them from surrounding particles (**Figure 3**) (45). The yield, purity, and size distribution of isolated EVs vary depending on the procedure (46). As a result, high-quality EVs must be isolated using a method that is acceptable for the study’s objectives and consistent with future analyses (47).

Separation/concentration can be carried out based on the EV’s biophysical parameters of size, density, charge, and surface composition (48). The optimum strategy is to use numerous approaches to optimize purity and yield (49). **Table 1** presents a comparison of EV isolation methods. The table includes essential features of various approaches, such as purity (ability to eliminate impurities), yield (number of EVs produced), scalability (capacity for large-scale applications), cost (total reagent and equipment expenses), and time (process length). Each criterion is categorized as Low, Medium, or High based on current research and practical concerns (50). The differences between EV isolation methods can be summarized by comparing their performance in terms of purity, yield, scalability, cost, and time.

**Table 1:** A comparison of extracellular vesicle (EV) isolation methods.

Method of isolation	Purity	Yield	Scalability	Cost	Duration
Ultracentrifugation	Moderate	High	Medium	Moderate	Long
Precipitation	Low	High	High	Low	Short
Size Exclusion Chromatography	High	Medium	Low	Moderate	Medium
Affinity-Based	High	Low	Low	High	Medium
Microfluidics	High	Medium	Low	High	Short

In terms of purity, size exclusion chromatography (SEC) and affinity-based approaches provide the highest levels of EV purity by effectively eliminating impurities such as proteins and lipoprotein (51). Ultracentrifugation (UC) achieves moderate purity but may co-isolate non-EV particles, whereas precipitation procedures produce the lowest purity due to the non-specific nature of polymer-induced precipitation, which can collect undesired proteins and other detritus (52). When it comes to yield, precipitation techniques and ultracentrifugation excel since they can process larger sample volumes and produce more EV amounts, albeit at the sacrifice of purity (53). SEC and micro-fluidics achieve moderate yields by selectively isolating EVs, whereas affinity-based approaches isolate specific EV subpopulations, resulting in lower total yields. Precipitation methods are the most scalable since they are simple and require little equipment, making them ideal for high-throughput applications (54). Ultracentrifugation and SEC are fairly scalable methods, but their time constraints and reliance on specialist equipment limit them (45). Affinity-based approaches and microfluidics, on the other hand, have low scalability due to sample volume limitations and the requirement for specialized reagents or devices (52). Precipitation methods are the most cost-effective since they require only basic reagents and equipment (49). Ultracentrifugation and SEC are somewhat expensive, including expenses incurred for equipment and consumables. The most expensive choices include microfluidics and affinity-based approaches, which require specialized reagents like antibodies and complex isolation devices.

The time necessary for isolation varies significantly (47). The fastest methods of isolation are precipitation and microfluidics, which take only a few hours to complete. SEC takes intermediate time for processing and elution; however, ultracentrifugation is the slowest procedure, requiring several hours to days due to the consecutive centrifugation processes (49). Overall, precipitation technologies are best suited for applications that require high throughput at cheap cost, where purity is less important. Ultracentrifugation strikes a balance between yield and purity, although it is labor-intensive and time-consuming (45). SEC provides excellent purity while maintaining EV integrity, making it ideal for delicate downstream applications. Affinity-based approaches are very specific to certain EV subpopulations, but they are expensive and difficult to scale. Microfluidics offers precision and speed but is restricted by its cost and sample volume capacity (55). The individual research goals, available resources, and expected downstream applications should all be considered when selecting an isolation strategy (50).

This comparative analysis demonstrates that no single isolation strategy is universally perfect; instead, the best methodology is strongly influenced by the study’s specific goals. For researchers who prioritize purity and EV integrity, particularly for downstream applications like proteomics or RNA analysis, SEC or affinity-based procedures may be the best option, but their yield or scalability might be limited. When large-scale EV synthesis is a top priority, particularly for diagnostic or therapeutic research, precipitation or ultracentrifugation may provide a more feasible cost-to-output ratio. Finally, a hybrid technique that incorporates complimentary methods may provide a personalized solution by optimizing both quality and efficiency based on the experimental situation.

## 6 Methods of extracellular vesicles characterization

To enhance comprehension of EVs’ functions and evaluate the related diagnostic and therapeutic tools, correct classification is essential. Each approach harbors different advantages and disadvantages regarding the interpretation of results (**Table 2**).

**Table 2:** Summary of Methods for Extracellular Vesicle (EV) Characterization.

Method	Key features	Advantages	Limitations	References
Dynamic Light Scattering (DLS)	Measures particle size using light scattering caused by Brownian motion.	Fast, sensitive for monodisperse suspensions, and measures particles from 1 nm to 10 µm.	Ineffective for polydisperse samples; larger particles obscure signals from smaller ones.	(56–62)
Nanoparticle Tracking Analysis (NTA)	Tracks Brownian motion of individual particles, combining laser light scattering with camera imaging.	Provides direct visualization, measures vesicles from 60 to 1,000 nm, and fluorescence tagging enhances specificity.	Does not differentiate EVs from contaminants without fluorescence labeling. Subject to photobleaching and additional purification requirements.	(63–67)
Flow Cytometry (FC)	Detects and characterizes EVs based on surface or cytoplasmic protein markers using light scattering.	High throughput, multiplex fluorescence for markers, and nanoscale FC can detect particles from 100 to 1,000 nm.	Conventional FC limited to particles >300 nm; requires rigorous calibration and validation to ensure accuracy.	(4, 56, 68–71)
Optical Microscopy	Uses fluorescent dyes for lipid membrane or nucleic acid visualization. Advanced techniques include STED.	Simple, enables phenotypic and biomarker analysis, and advanced techniques achieve up to 16 nm resolution.	Limited resolution for basic microscopy (200–300 nm). Autofluorescence and photobleaching pose challenges for analysis.	(72–75)
Transmission Electron Microscopy (TEM)	High-resolution imaging (<1 nm) of EV size and morphology; Cryo-TEM preserves native structure.	Allows precise EV characterization, distinguishes vesicles from contaminants, and immunogold labeling enables phenotyping.	Preparation artifacts can create “cup-shaped” appearances. Cryo-TEM requires advanced equipment and expertise.	(76–82)
Scanning Electron Microscopy (SEM)	Provides surface imaging using backscattered electron signals; ESEM reduces sample preparation artifacts.	High-resolution surface characterization and effective for environmental EV studies.	Lower resolution for ESEM; preparation artifacts and surface coating may affect accuracy.	(83–85)
Atomic Force Microscopy (AFM)	Non-invasive 3D imaging with sub-nanometer resolution for morphology and mechanical property analysis.	Non-destructive; analyzes EVs in native states; allows subsequent analyses with additional techniques.	Results sensitive to experimental conditions, such as probe state and interaction with sample.	(86–89)
Combined Reflectance and Fluorescence Confocal Microscopy	Integrates reflectance for precise focusing and fluorescence for marker identification.	Cost-effective; allows rapid screening for marker colocalization and isolation efficiency.	Bias toward larger EVs; requires complementary methods like SEM for smaller vesicle confirmation.	(57)

DLS, Dynamic Light Scattering; NTA, Nanoparticle Tracking Analysis; FC, Flow Cytometry; STED, Stimulated Emission Depletion; TEM, Transmission Electron Microscopy; Cryo-TEM, Cryogenic Transmission Electron Microscopy; SEM, Scanning Electron Microscopy; ESEM, Environmental Scanning Electron Microscopy; AFM, Atomic Force Microscopy.

### 6.1 Dynamic light scattering

Dynamic light scattering (DLS) is also known as photon correlation spectroscopy (PCS) which determines the size of the particle by measuring fluctuations of the scattered light caused by the Brownian movement of the particles in the solution (56). DLS is fast and highly sensitive for uniform suspensions because unlike imaging-based methods, DLS uses photon detectors to analyze the entire sample (60). This enables it to analyze particles from 1 nm to 10 µm without extensive preprocessing (90).

### 6.2 Nanoparticle tracking analysis

EV research has increasingly relied on nanoparticle tracking analysis (NTA) to quantify particle size and concentration. NTA tracks the Brownian motion of individual particles suspended in a solvent and uses the Stokes Einstein equation to calculate their hydrodynamic diameter (63–66). Further, EV subpopulations have been studied using fluorescent NTA. Thus, labeled surface markers or RNA-targeting molecular beacons have been used to stain particular vesicle subtypes and RNA cargo. These advancements have allowed insight into the diversity and function of EVs (64, 91, 92).

### 6.3 Flow cytometry

Flow cytometry (FC) is a common method used to detect and characterize EVs by their surface or cytoplasmic protein markers (4, 68, 69). Typically, traditional flow cytometers can measure relatively large EVs, with diameters almost always greater than 300 nm (69). This method involves passing a focused stream of the fluid carrying particles through a laser beam of a specific wavelength and strategically placed visible and fluorescent light detectors measuring scattered light (56, 93, 94). The forward scattered light (FSC), detected in the path of the laser, gives information on particle size, while side scattered light (SSC), perpendicular to the beam, is a measure of the internal complexity of the particles, such as granularity (70). Nevertheless, conventional FC suffers from low sensitivity and resolution, which limits its ability to detect smaller EVs, e.g. those below 300 nm (56, 69, 71, 95).

This challenge has recently been addressed through several advancements. This technology linearly detects particles from 100 to 1,000 nm (small to large EV) and allows for multiplexed fluorescent detection (96, 97). Nano-flow cytometry (nFC) labels EVs with disease specific markers and gives insights into cellular origin and pathological state (52, 96, 97).

### 6.4 Optical microscopy

Optical microscopy provides a straightforward method for observing EVs due to their relatively large size, despite the diffraction-limited resolution of approximately 200 to 300 nm. However, unless combined with sophisticated methods like stimulated emission depletion (STED) microscopy, confocal microscopy, or total internal reflection fluorescence microscopy (TIRFM), optical microscopy is insufficient for revealing finer structural detail of smaller EVs, i.e., exosomes (72, 73). Resolutions of up to 16 nm are obtained with STED microscopy, which is suitable for EV characterization and morphological studies (74, 75, 98).

### 6.5 Transmission electron microscopy

Transmission electron microscopy (TEM) is the gold standard for EV imaging because it allows for imaging of single EVs with resolutions <1 nm (76). Cryo-EM is used to overcome these artifacts in order to render native EV structures with more accuracy without chemical fixation or dehydration (77, 78). This method is especially useful for characterizing the three-dimensional architecture of EVs, differentiating them from other contaminants (99–101). TEM can be further enhanced by immunogold labeling to identify specific EV proteins using gold conjugated antibodies for EV phenotyping in complex media (79, 102, 103). Another electron microscopy technique is scanning electron microscopy (SEM). High resolution images of EV surfaces are obtained using SEM by utilizing backscattered electron signals to distinguish between sample components (83). Samples may be sputter coated with metallic or carbon layers to provide contrast enhancement for surface characteristic study (84, 104, 105). The study of environmental surface degradation of EVs and their interaction with other materials has been instrumental using SEM (106).

### 6.6 Atomic force microscopy

Atomic force microscopy (AFM) is a non-destructive way to characterize EVs with three dimensional topographical images, providing details of their morphology and sub-nanometer resolution (86, 107, 108). AFM obtains detailed insight into their morphology, size, mechanical properties and surface charge by scanning a sharp tip over the EV surface (86, 87, 109). Moreover, since its non-destructive approach it allows for further characterization by subsequent techniques (110). AFM’s utility in its ability to analyze EVs under physiological state without staining or fixation (86, 88). Specific antibody coated surfaces are then applied to further enable the identification of EV subpopulations (89).

### 6.7 Combined reflectance and fluorescence confocal microscopy

Reflectance and fluorescence confocal microscopy integration is a practical and cost-effective way to characterize EVs. Using this approach, EV fluorescence signals are differentiated from nonspecific artifacts, leaving phenotypic characterization possible using standard confocal laser scanning microscopes. Using high intensity reflection planes from the coverslip and glass slide as references, reflectance microscopy allows for sharp focusing on EVs. Using this setup, lateral and axial resolutions of 198 nm and 492 nm, respectively, are achieved and allow visualization of EVs and their aggregates (57).

When put together, these characterization techniques provide wide tools for examining EVs at a variety of resolution, sensitivity, and molecular detail levels. While TEM and AFM provide excellent structural insights, tools like NTA and flow cytometry allow for quantitative and phenotypic research. Each method has unique strengths and limits, and when used together, they frequently give the most thorough insight. As the field progresses, integrated techniques that combine high-resolution imaging and molecular profiling are anticipated to play an important role in improving EV-based diagnoses and therapies. The biological inquiry and the study’s technical constraints ultimately determine the best method or combination to use.

## 7 Usage of extracellular vesicles in cancer diagnostics

EVs have emerged as promising biomarkers for cancer diagnostics. These EVs can be isolated from various biofluids, such as plasma, bronchoalveolar lavage fluid (BALF), urine, and cerebrospinal fluid (CSF) (52), allowing a selective and non-invasive diagnostic approach for cancer detection. Among these, plasma-derived EVs are the most commonly used biofluid due to their accessibility and the presence of a diverse range of EVs containing tumor-specific markers (111, 112).

EVs possess the ability to carry a variety of biomarkers with significant diagnostic and prognostic potential. Tumor-associated miRNAs are frequently upregulated in cancer patients and have been studied for their role in early cancer detection and monitoring therapeutic responses. The integration of multiple biomarker types—genetic, protein, and lipid—into EV analysis holds promise for providing a more comprehensive approach to cancer diagnostics (113). However, challenges remain, such as variability in EV concentrations across individuals and different biofluids, which must be addressed to enhance diagnostic accuracy (114).

### 7.1 Breast cancer

In breast cancer, specific EV-associated miRNAs have demonstrated strong diagnostic potential. Elevated levels of miR-21 and miR-155 in EVs are linked to tumor aggressiveness, immune interactions, and metastasis. Conversely, reduced levels of miR-126 in BRCA mutation-positive patients suggests potential for personalized diagnosis (115). Similarly, EVs carrying HER2 and EGFR provide valuable non-invasive tools for monitoring tumor status and tracking treatment response, making them essential for improving breast cancer detection and treatment personalization (116). Furthermore, multi-miRNA diagnostic panels have been demonstrating improved accuracy over single biomarker approaches. For example, a four-miRNA panel (miR-1246, miR-206, miR-24, and miR-373) achieved 98% sensitivity and 96% specificity, significantly outperforming individual markers and offering an enhanced breast cancer diagnostic tool (117). Additionally, miR-10b and miR-639 have been found to promote tumor invasiveness, migration, and epithelial-mesenchymal transition in breast cancer stem cells. These traits make them potential biomarkers for early diagnosis, with sensitivity and specificity ranging from 64.8% to 83.3% (118, 119) (**Table 3**).

**Table 3:** EVs diagnostic biomarkers in different types of cancer EVs diagnostic biomarkers in different types of cancer.

Cancer	Biomarker	Category	Sensitivity (%)	Specificity (%)	References
Breast	miR-21-5P	miRNA	86.7	93.3	(120)
Breast	miR-155	miRNA	93	85	(121)
Breast	miR-127	miRNA	88.1	86.2	(122)
Breast	miR-451 *	miRNA	93	79.3	(122)
Breast	miR-148a *	miRNA	56.1	78.9	(122)
Breast	miR-9-5p	miRNA	85.2	93.7	(123)
Breast	miR-148a-3p	miRNA	86.6	87.5	(123)
Breast	miR-17-5p	miRNA	70.6	65.2	(123)
Breast	miR-10b	miRNA	78.3	69.5 – 100	(119, 124)
Breast	miR-639	miRNA	65	–	(119, 124)
Breast	miR-142-3p	miRNA	68.5	73.7	(125)
Breast	miR-125b	miRNA	82	77	(121)
Breast	miR-148a-3p	miRNA	82.53	64.71	(126)
Breast	miR-181a	miRNA	85.54	61.76	(126)
Breast	miR-34a-5p	miRNA	76.53	83.53	(126)
CRC	hsa-miR-340-5p	miRNA	100	100	(127)
CRC	hsa-miR-19b-3p	miRNA	93.3333	100	(127)
CRC	hsa-miR-19a-3p	miRNA	93.3333	100	(127)
CRC	hsa-26a-5p	miRNA	93.3333	100	(127)
CRC	hsa-miR-21-5p	miRNA	93.3333	100	(127)
CRC	hsa-miR-145-3p	miRNA	93.3333	100	(127)
CRC	hsa-miR-330-5p	miRNA	93.3333	100	(127)
CRC	hsa-miR-26b-5p	miRNA	100	90	(127)
CRC	hsa-let-7f-2-3p	miRNA	100	90	(127)
CRC	hsa-let-7b-3p	miRNA	100	90	(127)
CRC	hsa-miR-576-3p	miRNA	100	90	(127)
CRC	hsa-miR-339-4p	miRNA	100	90	(127)
CRC	hsa-miR-15b-3p	miRNA	86.6667	100	(127)
CRC	hsa-miR-484	miRNA	86.6667	100	(127)
CRC	hsa-miR-339-5p	miRNA	86.6667	100	(127)
CRC	hsa-miR-374a-3p	miRNA	93.3333	90	(127)
CRC	hsa-miR-501-3p	miRNA	93.3333	90	(127)
CRC	hsa-miR-425-5p	miRNA	93.3333	90	(127)
CRC	hsa-miR-30e-5p	miRNA	100	80	(127)
CRC	hsa-miR-155-5p	miRNA	100	80	(127)
CRC	hsa-miR-425-3p	miRNA	80	100	(127)
CRC	hsa-miR-150-3p	miRNA	86.6667	90	(127)
CRC	hsa-miR-186-5p	miRNA	86.6667	90	(127)
CRC	hsa-miR-181a-2-3p	miRNA	86.6667	90	(127)
CRC	miR-23a	miRNA	89	70	(128, 129)
CRC	miR-301a	miRNA	89	70	(128, 129)
CRC	miR-92b	miRNA	80	80	(128, 130)
CRC	miR320d	miRNA	63.3	91.3	(128, 131)
CRC	miR-27a	miRNA	81.82	90.91	(128, 132)
CRC	miR-130a	miRNA	69.32	100	(128, 132)
CRC	miR-122	miRNA	89	89	(128, 133)
CRC	miR-99b-5p	miRNA	32.1	90.8	(128, 134)
CRC	miR-150-5p	miRNA	75.1	58.8	(128, 134)
CRC	miR-361-3p	miRNA	–	–	(135)
Pancreatic	miR-196a	miRNA	81	81	(136)
Pancreatic	miR-1246	miRNA	73	73	(136)
Pancreatic	ExmiR-191	miRNA	64.3	79	(137)
Pancreatic	ExmiR-21	miRNA	75.9	81	(137)
Pancreatic	ExmiR-451a	miRNA	62.1	85.7	(137)
Pancreatic	miR-1246	miRNA	66.7	100	(138)
Pancreatic	miR-4644	miRNA	75	76.9	(138)
Pancreatic	miR-210	miRNA	42	73	(139)
Pancreatic	miR-155	miRNA	53	78	(139)
Pancreatic	miR-19b	miRNA	85.48	90.57	(140, 141)
Pancreatic	miR-483-3p	miRNA	85.7	72.7	(140, 142)
Pancreatic	miR-3940-5p/miR-8069 ratio	miRNA	58.1	89.2	(140, 143)
Pancreatic	GPC1	Protein	100	100	(140, 144)
Pancreatic	ALIX	Protein	53.1	83.9	(140, 141)
Pancreatic	c-Met	Protein	70	85	(140, 145)
Pancreatic	PD-L1	Protein	14	94	(140, 146)
Prostate	miR-9-5p	miRNA	–	–	(147, 148)
Prostate	miR-196a	miRNA	89	100	(147, 149)
Prostate	lincRNA-p21	lincRNA	67	63	(147, 150)
Prostate	lincRNA-p21/PSA	lincRNA	53	94	(147, 150)
Prostate	miR-1246	miRNA	75	100	(151)
Prostate	Transmembrane protein 256	Protein	94	100	(151)
Prostate	Adipogenesis regulatory factor	Protein	81	100	(151)
Prostate	Ragulator complex protein LAMTOR1	Protein	81	100	(151)
Prostate	Plastin-2	Protein	75	100	(151)
Prostate	Ras-related protein Rab-2A	Protein	75	100	(151)
Prostate	Ras-related protein Rab-3B	Protein	75	100	(151)
Prostate	Ras-related protein Rab-3D	Protein	75	100	(151)
Prostate	Ras-related protein Rab-7a	Protein	75	100	(151)
Prostate	V-type proton ATPase 16 kDa proteolipid subunit	Protein	75	100	(151)
Prostate	Metalloreductase STEAP4	Protein	69	100	(151)
Prostate	Protein DJ-1	Protein	69	100	(151)
Prostate	Protein S100-P	Protein	69	100	(151)
Prostate	Synaptotagmin-like protein 4	Protein	69	100	(151)
Prostate	ADP-ribosylation factor-like protein 8B	Protein	63	100	(151)
Prostate	Proton myo-inositol cotransporter	Protein	63	100	(151)
Prostate	Ras-related protein Rab-6A	Protein	63	100	(151)
Prostate	Tetraspanin-6	Protein	63	100	(151)
Prostate	Claudin-10	Protein	56	100	(151)
Prostate	Claudin-2	Protein	56	100	(151)
Prostate	Claudin-3	Protein	56	100	(151)
Prostate	GDP-mannose 4.6 dehydratase	Protein	56	100	(151)
Prostate	Glucosamine-6-phosphate isomerase 1	Protein	56	100	(151)
Prostate	Lysosome membrane protein 2	Protein	56	100	(151)
Prostate	Major facilitator superfamily domain-containing protein 12	Protein	56	100	(151)
Prostate	Melanophilin	Protein	56	100	(151)
Prostate	Sepiapterin reductase	Protein	56	100	(151)
Prostate	Thioredoxin domain-containing protein 17	Protein	56	100	(151)
Prostate	3-hydroxybutyrate dehydrogenase type 2	Protein	50	100	(151)
Prostate	Calmodulin	Protein	50	100	(151)
Prostate	Carboxypeptidase Q	Protein	50	100	(151)
Prostate	Flotillin-2	Protein	50	100	(151)
Prostate	Galectin-3-binding protein	Protein	50	100	(151)
Prostate	P2X purinoceptor 4	Protein	50	100	(151)
Prostate	Protein dopey-2	Protein	50	100	(151)
Prostate	Protein S100-A6	Protein	50	100	(151)
Prostate	Ras-related protein Rab-35	Protein	50	100	(151)
Prostate	Serine/threonine-protein phosphatase 2A catalytic subunit alpha isoform	Protein	50	100	(151)
NSCLC	miR-21, miR-210, miR-1290	miRNA	77	83	(152)

miR, microRNA; miRNA, microRNA; ExmiR, Exosome-derived microRNA; lincRNA, long intergenic non-coding RNA; CRC, Colorectal Cancer; NSCLC, Non-Small Cell Lung Cancer.

### 7.2 Lung cancer

Studies identified differentially expressed miRNAs (DEMs) in plasma-derived small EVs (sEVs), such as miRNA-483-3p, upregulated in small cell lung cancer (SCLC), and miRNA-152-3p and miRNA-1277-5p, upregulated in non-SCLC (153). These DEMs regulate key biological processes, such as cAMP signaling and leukocyte transendothelial migration, aiding in early detection of SCLC and NSCLC. Additionally, miR-1246b found in BALF-derived EVs distinguishes malignant pulmonary nodules, while miR-505-5p in LA patients promotes tumor growth and inhibits apoptosis by targeting TP53AIP1 (118, 154). Furthermore, EGFR T790M/L858R-mutant non-SCLC cells produce EVs that accelerate the tumor growth by enhancing invasion, migration, and proliferation. Additionally, they complement EV-based diagnostic techniques by improving EGFRvIII mutation detection in circulating EV-RNA (152, 155). However, further research is required to refine and standardize these methodologies for clinical application.

### 7.3 Cervical cancer

EV-based biomarkers like squamous cell carcinoma antigen (SCCA) and miRNAs, such as miR-21, have been shown to be crucial for cervical cancer pathogenesis and progression. Notably, miR-486-5p has been shown to be overexpressed in cervical cancer and targets PTEN, activating the PI3K/Akt signaling pathway. This leads to enhanced cancer cell proliferation and growth, making miR-486-5p a potential biomarker for both cancer diagnosis and targeted therapy (156). Furthermore, proteomic analyses of serum-derived EVs from cervical cancer patients’ and healthy controls’ revealed 17 expressed proteins involved in metabolic processes and angiogenesis via the VEGF signaling pathway, including COX5A, IPO5, and ERI3 (157). Similarly, 19 upregulated proteins were associated with chemokine signaling pathways and increased cellular and metabolic regulation, highlighting their potential as therapeutic targets (157).

### 7.4 Ovarian cancer

EV-based biomarkers have also demonstrated promise in ovarian cancer detection. The miR-200 family, particularly miR-200a and miR-200c, is overexpressed in various ovarian cancer subtypes and associated with advanced disease stages and worse tumor-grades and survival outcomes. Furthermore, miR-200a has shown to have a strong correlation with tumor stage, grade, and lymph node metastasis, indicating its potential for early detection and prognosis (117). In addition to miR-200, recent studies show the enhanced diagnostic potential of exosomal miR-223. Where in combination with CA-125, demonstrated an increased sensitivity and specificity compared to CA-125 alone in the diagnosis of epithelial ovarian cancer patients, enhancing early-stage detection accuracy (158).

### 7.5 Hepatocellular carcinoma

Studies have demonstrated high diagnostic potential of combined EV-RNA and EV-surface antigen models for hepatocellular carcinoma (HCC), with sensitivity and specificity reaching 93.8% and 74.5% respectively. Similarly, combinations of serum-based EVs containing miR-21-5p, miR-92a-3p, and AFP show sensitivity of 95% and 86%, respectively. EV-lncRNA biomarkers like LINC00853 and lnc85 show superior diagnostic performance, surpassing serum AFP in sensitivity and specificity, making them effective for early HCC detection in high-risk groups. However, their ability to differentiate HCC from CCA remains limited, highlighting the need for EV-derived biomarkers (159). Furthermore, exosomal miRNAs play a crucial role in HCC diagnosis, metastasis, and disease progression. Where upregulated levels of miR-21, miR-221, and miR-222, and downregulated levels of miR-122, miR-145, and miR-199-a, shown to influence tumor growth, treatment resistance, and early cancer detection (160). EVs carrying IncRNA biomarkers have also shown the potential in enhancing HCC diagnosis, however, their low abundance requires highly sensitive detection methods (161). Moreover, a more recent study demonstrated the diagnostic potential of EV-based surface proteins for HCC, with the study showing significant differences in expressions observed between HCC patients and individuals with non-malignant liver disease (NMLD) (162). Furthermore, urinary EV glycoproteins, including 756 N-glycopeptides and 107 N-glycoproteins have been identified as potential non-invasive biomarkers for diagnosing HCC (163). Additionally, another study reveals six potential biomarkers for HCC, including EV-lncRNAs such as, DLEU2, HOTTIP, MALAT1, NEAT1, SNHG1, and TUG1. Among these biomarkers, TEV-MALAT1 showed good diagnostic ability for early-stage HCC, even in AFP-negative cases (164).

### 7.6 Colorectal cancer

Ren et al. discovered that exosomes secreted by colorectal cancer (CRC) cells, such as SW480 and HCT116, promote cell growth by activating STAT3 signaling under hypoxia conditions (128, 165). Similarly, Li et al. found that hypoxic EVs containing miR-361-3p increase tumor growth and block apoptosis by targeting TRAF3, activating the noncanonical NF-κB pathway (135). Accordingly, elevated levels of this miRNA in circulating exosomes are connected to poor prognosis and may serve as a potential marker and therapeutic target in CRC, as it is induced by hypoxia-inducible factor 1-alpha (HIF-1α) (135). Moreover, Liu et al. found that exosomes produced from CRC cells, such as mi-R106b-3p, stimulate cell invasion, migration, and the epithelial-to-mesenchymal transition (EMT) (166). Karimi et al. collected blood samples from CRC patients and showed higher expressions of exosomal miR-23a and miR-301a than normal controls with 89% and 70% sensitivity and specificity, respectively (129). Min et al. studies on individuals with CRC retain plasma EVs with distinct miRNA profiles, including Let-7b-3p, miR-1339-3p, miR-150-3p, and miR-145-3p, suggesting a new biomarker category for early diagnosis (127).

### 7.7 Pancreatic cancer

CA 19-9, a widely used diagnostic biomarker for pancreatic cancer, has limitations like low sensitivity, false negatives in Lewis negative phenotype, and higher false positive rates with obstructive jaundice, making early detection challenging (167). Recent studies have focused on the function of exosomes in relation to its potential diagnostic abilities in pancreatic cancer (168–170). For example, Li et al. revealed that exosomal miR-222 in pancreatic ductal adenocarcinoma (PDAC) promote invasion and proliferation of tumor cells through regulating p27 (168). Goto et al.’s study found three ExmiRs, ExmiR-191, -21, and -451a, outperformed CA 19–9 in early PC diagnosis, with sensitivity and specificity values exceeding 80% (**Table 3**) (167). Moreover, exosomes from pancreatic cancer patients overexpressed Glypican-1 (GPC1), suggesting its potential as a biomarker for pancreatic cancer diagnosis and stratification (140, 144).

### 7.8 Prostate cancer

Although prostate specific antigen (PSA) is the most used biomarker for the detection of prostate cancer, it lacks in its ability to stratify patients with high risk for early detection or with those with indolent prostate cancer (150, 171). Exosomes cargo that is distinct to prostate cancer can serve as a noninvasive tool for diagnosis (147, 172). For example, Logozzi et al. used PSA carried by exosomes (Exo-PSA) and showed superior performance than conventional PSA with almost 100% specificity and sensitivity (173). In such context of diagnostics, ExosomeDx, a pioneering company in oncology and precision medicine, uses urine-derived exosomal RNA in their flagship product, the ExoDx™ Prostate Test, to aid clinical decision-making (174, 175). Such actions encourage industries that bench to clinical shift can be possible for a therapeutic change.

Looking ahead, one could conclude from these findings that individualized therapeutic approaches in breast cancer, where miRNA panels like miR-1246, miR-206, and others demonstrate exceptional diagnostic accuracy, are among the most promising treatments associated with EV-based biomarkers (117). This opens the door to more specialized and efficient treatments. It is also a crucial area for clinical development because miR-200a-targeting ovarian cancer treatments, when combined with CA-125, offer improved early-stage diagnosis accuracy (176). Using a combination of exosomal miR-21 and miR-92a-3p biomarkers, hepatocellular carcinoma (HCC) exhibits encouraging findings with high sensitivity and specificity for early detection and distinction from non-malignant liver disorders (159). These advancements demonstrate how using EV-based biomarkers might enhance diagnostic skills and result in more individualized and successful treatment plans for a variety of malignancies.

## 8 Therapeutic uses of extracellular vesicles in cancer

### 8.1 EVs role in cancer immunotherapy

Immunotherapy, including cancer vaccines and immune checkpoint inhibitors, has emerged as a promising therapeutic regimen for cancer patients. Emerging as a promising adjunct to this domain in cancer management are the role of EVs, offering promising advantages such as precise targeting and improved immune responses (177, 178). Several studies have explored the role of EVs in enhancing drug-mediated apoptosis in cancers. For example, Cho et al., devised genetically engineered EVs derived from human CD8+ T cells incorporated with interleukin-2 and cetuximab against A549 lung cancer cells. The engineered EVs demonstrated greater cytotoxicity and enhanced susceptibility to immune-mediated cell death in the lung cancer cells. Additionally, the EVs exhibited EGFR- dependent targeting, which suggests their potential as precise and effective immunotherapeutic strategy for lung cancer (179). Tumor necrosis factor-related apoptosis-inducing ligand (TRAIL) is a promising anticancer treatment that preferentially binds to DR5 to cause apoptosis in cancer cells with no systemic effects. However, tumor resistance poses numerous therapeutic hurdles for TRAIL-based medications (180). To counter the challenges with the clinical use of TRAIL-based agents, many studies explored the use of EVs to improve outcomes. For example, exosomes engineered with TRAIL and loaded with triptolide (TRAIL-Exo/TPL) have demonstrated significant therapeutic potential in malignant melanoma. TRAIL-Exo/TPL enhanced tumor targeting, cellular uptake, apoptosis induction with reduced drug toxicity *in vivo* (181). Similarly, Qiu et al., developed mesenchymal stem-cell derived exosomes (MSCT-EXO) loaded with cabazitaxel (CTX) and TRAIL targeted towards oral squamous cell carcinoma (OSCC). By preventing the PI3K/Akt/mTOR pathway from being activated, the exosomes containing CTX and TRAIL showed synergistic antitumor effects and triggered apoptosis, which resulted in a considerable tumor suppression and tumor volume decrease (182). EVs also modulate radiation resistance, especially in oral squamous cell cancer. For instance, Chen et al. revealed that hypoxic cancer cell exosomal miR-340-5p targets KLF10/UVRAG, driving radioresistance in OSCC, making it a promising biomarker for theranostics (183). Furthermore, metformin reversed this impact by restoring KLF10 expression, making it a promising OSCC radiosensitivity therapy (183). In lung cancer, EVs loaded with TRAIL and dinaciclib (EV-T-Dina) were seen to downregulate anti-apoptotic factors such as cFLIP, MCL-1, and Survivin. Moreover, this combination when nebulized, demonstrated lower drug resistance and higher stability and efficacy in the treatment of lung cancer (184). These studies demonstrate the potential of EVs to modulate apoptosis in cancers by improving the efficacy of drug-induced apoptosis and reducing drug resistance.

Additionally, some research has demonstrated that EVs can be used to boost the effectiveness of different immune activation drugs, especially those that trigger the Stimulator of Interferon Genes (STING) pathway. To increase CDN efficacy, Jang et al. designed an EV that contained the cyclic dinucleotide (CDN) STING agonist ExoSTING. By specifically targeting antigen-presenting cells inside the tumor microenvironment, ExoSTING was able to boost immune activation by encouraging local Th1 responses, CD8+ T cell recruitment, and systemic anti-tumor immunity against the tumor. This effect minimized systemic inflammation and improved CDN efficacy (185). Similarly, McAndrews et al., devised an engineered exosome, iExoSTINGa, that delivered the STING agonist cyclic GMP-AMP, that demonstrated enhanced targeting efficiency and pharmacokinetics compared to free STING agonists (186).

Exosomal PD-L1 has also emerged as an important factor in tumor immune evasion. Tumor-derived small EVs (TDSEVs) high in PD-L1 block T cell activation in draining lymph nodes, contributing to resistance to immune checkpoint inhibitors (187, 188). Interestingly, CRISPR/Cas9-mediated deletion of Rab27a or neutral sphingomyelinase-2 (nSMase2) decreased exosomal PD-L1 levels where nSMase2 deletion further reduced total PD-L1 without changing surface expression (187). Therefore, blocking exosomal PD-L1 dramatically decreased tumor growth, even in resistant mice, and worked in tandem with anti-PD-L1 antibodies, highlighting its potential as a new immunotherapeutic target (188).

A promising substitute for other immunotherapies is the therapeutic cancer vaccination. By exposing dendritic cells (DC) to tumor-specific antigens, cancer vaccines improve adaptive immunity and encourage long-lasting T cell responses (189). EVs are promising candidates for cancer vaccines against a variety of malignancies, including malignant melanoma, according to numerous research. For instance, by promoting DC maturation and elevating CD8+ T cells and serum interferon α (INF-ƛ), melanoma cell-derived EVs suppressed tumor growth and metastasis and produced anti-tumor immunity (190). Ma et al. demonstrated a similar effect using Melanoma tumor cell-derived microparticles (T-MPs). The T-MPs worked to activate a lysosomal pathway in DCs and thus facilitating tumor antigen presentation to CD8+ T cells (191). These studies show the potential of EVs in enhancing immune functions and their potential for clinical use.

### 8.2 EVs as chemotherapy-delivery vehicles in cancer management

The use of EVs as drug delivery vehicles has emerged due to their natural biocompatibility, ability to traverse biological barriers, and inherent targeting capacity. Unlike traditional synthetic delivery systems, EVs have been shown to provide a safer and more efficient alternative, capable of reducing systemic toxicity and enhancing therapeutic efficacy of agents in the management of various cancers (192, 193). EV-based drug delivery vehicles showed promising potential in treating triple-negative breast cancer (TNBC). Macrophage-derived EVs loaded with paclitaxel and doxorubicin (Dox) showed effective accumulation in TNBC cells with significant anti-proliferative effects, offering a novel strategy in addressing the challenges of treating cancers such as TNBC (194). Furthermore, various studies explore exosomes as potential drug delivery agents. Exosomes show promising natural nanoparticles for drug delivery, particularly in multidrug- resistant cancers. Kim et al. assessed the effect of macrophage-derived exosomes loaded with paclitaxel (exoPTX) in murine lung carcinoma model. EVs have the potential to be effective drug delivery vehicles for treatment-resistant malignancies, as evidenced by the study’s 50-fold enhanced cytotoxicity and notable anticancer effects (195). Additionally, exoPTX demonstrated completely accumulated in pulmonary metastatic cells without affecting normal tissue. These findings highlight the potential clinical benefits, as this delivery model could reduce systemic toxicity seen with conventional chemotherapy by selectively targets cancer cell and bypassing drug resistance mechanisms (195). In addition, compared to free Dox, another study created Exo-Dox, a mixture of Dox and exosomes produced from mesenchymal stem cells, to target osteosarcoma *in vitro*. Exo-Dox demonstrated less cardiotoxicity, improved anti-tumor effectiveness, and increased cellular absorption (196). Moreover, exosomes loaded with gemcitabine (ExoGEM) demonstrated similar findings against pancreatic cancer cells. ExoGEM demonstrated enhanced targeting and cytotoxicity compared to systemic gemcitabine, selectively increasing drug concentration at tumor sites with minimal systemic effects. ExoGEM was also seen to suppress tumor growth, prolong survival, and completely eradicate tumors in tumor-bearing mice (197). Interestingly, exosomes have been shown to demonstrate the ability to deliver chemotherapy to target brain cancer. In a zebrafish brain cancer model, brain endothelial cell-derived exosomes enhanced drug uptake via receptor-mediated endocytosis and effectively crossed the blood-brain barrier to target the brain. This method was shown to significantly reduce tumor growth supported by a reduction in the fluorescent markers of cancer cells, highlighting the utility of exosome-based delivery systems in targeting brain cancers (198). These findings highlights the potential clinical utility of exosome-based drug delivery systems, which could offer treatment options for brain cancers that were previously considered untreatable with conventional chemotherapeutics (198).

Furthermore, multiple studies explored the prospect use of functionalized exosomes with aptamers for targeted therapy. For example, Bagheri et al. devised an engineered exosome-based delivery system for Dox in a murine colon adenocarcinoma model (21, 21). Mesenchymal stem cell-derived exosomes encapsulated Dox and were functionalized with MUC1 aptamers for targeted delivery to MUC1-positive cancer cells. The exosomes demonstrated superior Dox transportation, enhanced accumulation in tumor cells, and enhanced clearance compared to free Dox. Additionally, the engineered exosomes significantly suppressed tumor growth in the adenocarcinoma model (199). These findings further support the important clinical implications of exosome-based delivery systems, as they can potentially reduce side effects and improve treatment outcomes in managing colorectal cancer (21). Another study also demonstrated similar potential clinical implications by exploring aptamer-functionalized exosomes loaded with Dox for colorectal cancer treatment. Another study explored aptamer-functionalized exosomes loaded with Dox for colorectal cancer treatment. The study functionalized Dox-loaded exosomes with AS1411 aptamers. The functionalized exosomes demonstrated enhanced cytotoxicity and significant tumor growth suppression through target accumulation and retention (200). These studies highlight the potential of functionalized exosomes as a safe and effective therapeutic strategy in cancer treatment. Moreover, other EVs such as nanovesicles have been shown to have promising drug delivery abilities. Jang et al. demonstrated that bioengineered exosome-mimetic nanovesicles loaded with chemotherapeutics outperformed traditional liposomal delivery systems. The nanovesicles demonstrated efficient tumor targeting and enhanced tumor cell death and reduced tumor growth with minimal adverse systemic effects (201).

Studies have been exploring the role of synthetic or engineered EVs in cancer treatment. Engineered EVs are bioengineered versions of naturally occurring EVs modified to enhance their therapeutic potential. These EVs are modified by surface modifications, cargo loading, or genetic alterations, offering greater biocompatibility to natural EVs (202). For example, engineered exosomes have been studied to reverse drug resistance in drug-resistant cancers. Liang et al. demonstrated that exosomes co-delivering 5-fluorouracil (5-FU) and a miR-21 inhibitor effectively reversed 5-FU-resistance in resistant colorectal cancer cells. As a possible biomarker for early identification and treatment response monitoring, EVs from plasma and bronchoalveolar lavage fluid are enriched with miR-21, a microRNA linked to non-small cell lung cancer (NSCLC) (203). Furthermore, Wang et al., showed that engineered mimic vesicles derived from erythrocyte membranes had the ability to overcome multidrug resistant tumors. The mimic vesicles were designed to co-deliver Dox and P-glycoprotein siRNA and achieved high drug loading rates able to effectively silence P-glycoprotein and enhancing Dox-induced tumor inhibition (204). Moreover, engineered macrophage-derived exosome-coated nanoparticles were shown to increase chemotherapy response in TNBC. This was done by modifying exosome surfaces with a c-Met-targeting peptide, which significantly increased tumor cellular uptake, targeting efficacy, and Dox-induced tumor inhibition, offering a promising strategy for TNBC treatment (205). These engineered EVs can hold extreme clinical potential by offering precise treatment while reducing off-target effects seen in conventional chemotherapy.

Proteolysis-targeting chimeras (PROTACs) serve as a new therapeutic approach to target select pathogenic proteins through hijacking the ubiquitin-proteasome system for protein breakdown (206, 207). Tumor-targeting PROTACs represent recent innovations that utilize receptors which specifically bind to overexpressed receptors found on cancer cells (208). Additionally, the development of “pro-PROTACs” represents a new approach for minimizing off-tumor toxicity by requiring tumor-associated enzymes to activate them (208). Nanoparticle-assisted delivery and PEGylation have also improved the stability, solubility, and pharmacokinetics of PROTACs, allowing for higher tumor accumulation and fewer systemic effects (209, 210). These engineered approaches have enabled scientists to generate specific potent and safe PROTAC candidates for treating breast and prostate cancers (211, 212).

These studies underscore the growing clinical potential of EVs as efficient drug delivery vehicles in cancer therapy. By enhancing drug targeting and availability, improving efficacy, and minimizing systemic toxicity, EV-based delivery systems present a promising alternative to conventional chemotherapeutic approaches. Additional studies exploring the use of EVs as drug delivery vehicles in clinical settings are needed to provide valuable clinical data.

## 9 Engineering therapeutic extracellular vesicles: preparation and storage challenges

### 9.1 Preparation strategies for therapeutic extracellular vesicles

EVs can inherently facilitate the transfer of macromolecules across cells, rendering them advantageous vehicles for drug delivery owing to their biocompatibility, and targeting efficacy. Drug incorporation into EVs can be accomplished via endogenous or exogenous techniques.

Endogenous loading is the process of incorporating therapeutic substances into parental cells, such as through gene transfection or co-incubation. For example, Chen et al. employed lentiviral transfection to overexpress miR-375 in adipose-derived MSCs, resulting in exosomes that stimulated bone repair (213). Co-incubation has also been used to successfully load hydrophobic medicines such as doxorubicin (214), paclitaxel (215) and curcumin (216), with Zhu et al. showing that paclitaxel-loaded MSC exosomes had increased antiproliferative properties (217).

On the other hand, exogenous loading introduces drugs directly into EVs post-isolation, typically achieving higher efficiency. Zhou et al. encompassed electroporation, utilized for siRNA delivery into EVs that was effective yet potentially leading to EV aggregation, which can be alleviated by the addition of EDTA as demonstrated by Kooijmans et al. (189, 218). Yerneni et al. used ultrasonication enhances drug loading by temporarily disrupting EV membranes, demonstrating the ability to reverse inflammation *in vivo* with curcumin-loaded EVs (219). Mechanical extrusion and freeze-thaw cycles have been also employed; however, they may compromise membrane integrity or exhibit reduced efficiency (220, 221).

Targeting can be improved by membrane alteration. For instance, Liu et al. used genetic engineering to alter EVs with RGD peptide for retinal treatment (222). Lee et al. developed exosomes that incorporated sodium azide-containing lipids and linked them to targeting peptides through copper-free click chemistry to improve the targeting of cancer cells (223) and Du et al. utilized ultrasonic drug loading and CD47 alteration to create EVs that could evade immune clearance and target tumors (224).

EV engineering methods should be chosen based on cargo type, therapeutic purpose, and targeting. Endogenous methods are gentler and retain EV integrity, making them suited for sensitive payloads, while exogenous methods are more efficient but may compromise EV stability (220, 225). Using gene transfection and ultrasonication together can improve multifunctionality and therapeutic performance. Membrane modification is useful for targeted administration but requires cytotoxicity and long-term biocompatibility testing (220).

### 9.2 Storage conditions and stability considerations

Efficient storage of EVs is essential for maintaining their structural integrity, molecular composition, and bioactivity, hence ensuring consistent particle count, cargo (protein/RNA), and morphology across diverse EV sources (226).

Proper storage of EVs is essential to maintain their structure, cargo integrity, and biological function (227). A systematic review by Ahmadian et al. analyzed 50 studies and found that −80 °C is the most reliable temperature for long-term EV preservation, effectively maintaining particle concentration, RNA/protein content, and morphology across various sources (227). However, repeated freeze-thaw cycles were shown to significantly damage EVs, reducing particle count, promoting aggregation, and degrading miRNA—up to 70% loss after a single cycle in one study (228). Short-term storage at 4 °C is acceptable for a few days, especially in native biofluids, but longer periods lead to protein degradation and membrane damage (229). Elevated temperatures (>25 °C) accelerate deterioration and are unsuitable for storage (39, 42, 56). The use of cryoprotectants, particularly trehalose, was shown to preserve EV morphology and function, including during freeze-drying (lyophilization), making it a promising strategy for maintaining stability without ultra-cold conditions (230, 231). Moreover, storage buffers like PBS alone are insufficient, often leading to aggregation, whereas formulations like PBS with human albumin and trehalose (PBS-HAT) significantly improve EV preservation (232). Storing EVs within their native biofluids, such as plasma or saliva, also enhances stability compared to purified suspensions (233). Interestingly, some findings suggest −80 °C may outperform liquid nitrogen (−196 °C) in preserving RNA content and membrane integrity (234).

Despite progress, standardized storage techniques for long-term EV preservation are still immature and require adjustment based on EV type and planned application (235). Key problems, such as heterogeneity, low yield, scalability, and stability, remain barriers to successful clinical translation (236, 237). Maintaining EV integrity during storage requires enhanced stabilization measures, such as freezing at -80°C (236, 237). Progress in EV characterization, imaging, and synthetic alternatives, together with enhanced technology and quality control, will be critical to the advancement of EV-based targeted therapeutics (236, 237).

### 9.3 *In Vivo* biodistribution and safety considerations of extracellular vesicles

#### 9.3.1 Circulation, biodistribution, and metabolic fate

EVs are natural endogenous cargo delivery vehicles (238). They have inherent targeting properties that allow for interaction with target cells. Many factors contribute to the homing of EVs *in vivo* including components of the EVs like surface markers and phospholipids, the cell source of the EVs and the administration route all of which discussed in this section.

#### 9.3.2 Role of surface markers and phospholipids in EV behavior

Surface markers and receptors on EVs serve multiple functions, including facilitating the targeting of certain cells and organs (239). For example, tetrasponins can form a complex with integrin α4 and CD49D to target CD54 endothelial cells and pancreatic cells (240, 241). This binding is pivotal in EV-mediated tumor development. Moreover, ligands such as transferrin (Tf), which are abundantly expressed on cancer cells, can bind to transferrin receptors (TfR) inherently present on EV surfaces, so enabling targeted delivery (241, 242). Interestingly, EVs generated from milk possess surface proteins that facilitate targeted absorption. For instance, Näslund et al. demonstrated that these connections facilitate the targeting of monocyte-derived dendritic cells (DCs) by EVs through the MUC1–DC-SIGN pathway (241, 243).

Beyond surface markers, the lipid composition of the EV membrane, especially phospholipids, plays a central role in directing EVs to specific cells. Phosphatidylserine (PS) can interact with PS receptors on immune cells, frequently resulting in the clearance of EVs by macrophages. For example, TIM4 binds PS to promote engulfment, while cloaking PS with annexin V reduced macrophage uptake by 66% (244). Furthermore, EV surface glycans can interact with cancer cells through the binding of CCR8 and CCL18 (245). These findings underscore the substantial impact of membrane composition on the targeting and uptake of EVs, which, if considered, will improve therapeutic applications.

#### 9.3.3 Influence of cell source on EV homing

The targeting of EVs is significantly determined by their cell of origin, as EVs typically exhibit a preference for homing to tissues or cells that are closely associated with their parent cells (236). For instance, endothelial EVs from the brain can accumulate in cerebral tissue (198), while melanoma EVs target melanoma metastases (246). EVs from mesenchymal stem cells (MSC-EVs) have increased accumulation in mice kidney with acute kidney injury, suggesting disease-specific biodistribution (247). Tumor-derived EVs exhibit a natural affinity for tumor sites, facilitated by specific surface molecules such as E-cadherin present on prostate cancer-derived EVs, which enable targeting of homologous tumors (248). Immune cell-derived EVs, particularly from DCs, exhibit MHC molecules that facilitate the targeting of immune cells, thereby augmenting immune responses (249). MSC-derived EVs demonstrate efficacy in targeting conditions such as type 2 diabetes and neurodegenerative disorders (250). Endothelial cell-derived EVs demonstrate a significant affinity for bone, indicating potential for bone-targeted therapies (251). This emphasizes the potential of utilizing the inherent homing abilities of EVs, influenced by their cellular origin, for targeted therapeutic applications in diverse conditions.

EVs can also passively target tumor sites due to enhanced permeability and retention (EPR) effect in the tumor microenvironment (TME) that is characterized by permeable vasculature facilitating the passive accumulation of EVs. A study has shown that EVs originating from melanoma cells possess a natural propensity for lung tissues, resulting in high accumulation in lung metastases (236). Multiple myeloma and breast cancer cells get miR-15a or miR-16 from bone marrow-derived MSC (BM-MSC) EVs, limiting proliferation and angiogenesis (252). In contrast, the activation of cancer cells generally directs stromal cell-derived EVs towards pro-tumor phenotypes. For instance, fibroblasts activated by hepatoma cells demonstrate a notable elevation of SPOCK1/testican-1 pathways, facilitating the advancement of hepatoma cells (253, 254). When fibroblasts are stimulated by cancer cells like hepatoma, they release EVs with tumor-promoting cargo (e.g., SPOCK1/testican-1), which tend to home back to tumor sites and support cancer progression (252, 254). MSC-derived EVs also promote tumor migration via affecting integrin expression and MET. MiR-374a-5p-loaded EVs of gastric cancer-derived MSCs target HAPLN1 to boost gastric tumor integrin expression and cell migration (255).

Because the functions of the EVs imitate their originating cells, EVs can also possess antitumor activity if released from immune cells like DCs, natural killer (NK) cells and macrophages. NK cells, intrinsic tumor eradicators inside the TME, have been investigated in many immunotherapeutic approaches including adoptive NK cell transfer, CAR-NK treatment, and checkpoint inhibition (254, 256). EVs derived from activated primary natural killer (NK) cells or NK-92 cells stimulated by IL-12, IL-15, and IL-18 exhibit superior capacity to infiltrate and target solid tumors in comparison to those from dormant NK cell lines (257). DCs derived EVs show increased accumulation in spleen (236), giving antigens to T lymphocytes to elicit their antitumor response (258). Consequently, DC-EVs are inherently connected to T cell functionality and can directly and indirectly boost T cell immunity (254, 259). M1 macrophage-derived EVs similarly induce tumor death by decreasing the expression levels of CCR4, Foxp3, and CTLA-4 in canine peripheral mononuclear cells cocultured with tumor cells (254, 260). While specific targeting of EVs is not fully understood, integrins are key molecules involved in organ-specific metastasis of tumor cells (236).

These findings show how the source and activation state of EV-producing cells affect their targeting (homing) and biological consequences, providing a solid foundation for future EV-based targeted therapeutics.

#### 9.3.4 Influence of administration routes on EV distribution and targeting

The route of administration of a drug influences its arrival rate to the target organ, its metabolism, and consequently the ultimate concentration achieved (261). Similarly, as EVs serve as vehicle for drug delivery, their mode of administration also affects the homing sites of these EVs (241).

Intravenous injection (IV) is the predominant route as it circumvents metabolism, hence preserving medication concentration (262). Wiklander et al. interestingly found that IV administered EVs accumulate in the liver more commonly than other routes by 60% (263). This was also emphasized by Zhou et al. who demonstrated that EVs tend to accumulate more in the liver and spleen through IV route compared to intraperitoneal (IP) injection where EVs seemed to accumulate more in adipose tissue broadly (264). Brossa et al. found out that administered human liver stem cells-derived EVs through the IV route inhibited subcutaneous tumors (265). However, IV injection poses problems such as off-target accumulation and rapid clearance rate (241, 266).

IP injections offer another viable approach. For example, Heidari et al. used MSC-derived EVs delivered intraperitoneally to treat acute colitis (267), while Nojehdehi et al. demonstrated immune-modulatory and glucose-stabilizing effects using adipose MSC-EVs via this route (268). This makes the IP route more suitable for metabolic diseases as EVs tend to accumulate in the pancreas (236, 263, 269).

Subcutaneous injections tend to accumulate in the digestive system (236), but face obstacles like adipose tissue and fibrovascular networks, affecting drug absorption. Research shows limited exosomes enter systemic circulation, with IP injections lasting longer due to reduced lymphatic flow (270).

The oral route is the favored approach because it is simple and does not require skilled staff for administration (271). Orally administered human breast milk-derived EVs have been demonstrated to mitigate gut inflammation by modulating immune responses (241, 272), specifically by promoting Treg and Th2 differentiation while inhibiting Th1 and Th17 cells (273). This indicates both direct and indirect effects on T cell-mediated immunity (272, 273). Therefore, this data suggests that oral route administration accumulates more in the small intestine which makes it suitable for digestive system diseases (241).

Local delivery, particularly intratumoral delivery, emphasizes targeting damaged tissue to enhance effectiveness of the injected therapy (274). In a study utilizing a prostate cancer mouse model, Rivoltini developed TNF-related apoptosis-inducing ligand (TRAIL)-armed exosomes for intratumoral injection. The results indicated an effective binding to tumor tissue, leading to a 58% reduction in tumor size (275). Although this method ensures efficient delivery and demonstrates a decrease in tumors, it is not desired by many patients due to its invasive nature and the possibility of an abrupt drug release that might be lethal (241, 276).

Betzer et al. investigated the impact of intranasal exosome injection. Interestingly, the study found that this approach efficiently accumulated in the brain, indicating their promise as a non-invasive treatment for neurological diseases (277). This finding demonstrate the possibility and effectiveness of intranasal delivery for targeted brain therapy.

As stated in the abovementioned routes, it is essential to correlate the targeted disease with the route of administration, as this relationship directly influences therapeutic potential. For example, IV administration is favored in systemic disorders, although there is a risk of off-targets (241), IP is more suitable for metabolic diseases (241, 269), and oral route targets more gastrointestinal diseases (241, 273). Among all, the local delivery is more likely the ideal choice of therapy as it offers fewer side effects, lower off-targets and augmented therapeutic effects (241).

Technical challenges such as rapid uptake and dye transfer hinder the understanding of EV clearance (278); however, recent studies employ that EVs have a short half-life, with blood levels influenced by secretion and rapid clearance (279).

### 9.4 Toxicity profiles of extracellular vesicles: the importance of standardized evaluation methods

EVs are involved in many physiological processes and diseases where they are generally considered safe, but not all have beneficial effects (280). For example, cancer-derived EVs can promote malignancy and may cause unexpected adverse reactions (280). Toxicity assessments conducted in rodents with EVs derived from MSCs, fibroblasts, HEK293T, and Expi293F cells indicated no significant adverse effects on hematological parameters, organ histopathology, or immune activation (280). However, mild inflammation in the liver and kidney was occasionally observed, though it did not result in significant immune toxicity (281). Furthermore, immunotoxicity studies indicated minimal immune responses, with certain EVs (e.g., derived from bovine milk) causing slight leukocyte proliferation and complement activation, whereas MSC-EVs exhibited largely inert characteristics (282). No instances of systemic anaphylaxis or significant cytokine responses were observed (283). Evaluations of gene toxicity through comet and micronucleus assays indicated no genotoxic effects from MSC- or milk-derived EVs (284). However, exosomes from chemo-resistant glioblastoma models demonstrated potential gene interaction (285). More importantly, concerns persist regarding tumorigenicity; MSC-exosomes facilitated cancer cell migration and colony formation, while exosomes derived from cancer sources enhanced tumor progression and established immunosuppressive microenvironments in various *in vivo* models (285–287). The findings highlight the necessity for comprehensive and standard guidelines for toxicity assessment to facilitate the safe clinical translation of EV therapies.

## 10 Future perspectives and current limitations

A number of cutting-edge technologies have been established to change cancer diagnostics to offer more accurate and non-invasive ways than those used for early detection (288). Phototherapy refers to the use of specific light wavelengths to activate therapeutic agents, such as photosensitizers, for the treatment of cancer (289). Current research in photodynamic therapy (PDT) has produced responsive photosensitizers which detect tumor environmental signals for precise targeting and decreased side effects in other tissues (290). Moreover, the use of nanotechnology in PDT enables better delivery and higher accumulation of photosensitizers in tumors, which improves treatment outcomes (291). These advancing technological innovations bring substantial progress to cancer phototherapy while creating paths for more accurate treatment methods with reduced invasiveness (292).

Another advances include the integration of nanotechnology to create biosensors and platforms for sensitive biomarkers detection that are exosome-based. For example, Patolsky et al. used Magnetically Amplified DNA Assays (MADA) to improve DNA detection by incorporating nucleic acid-modified magnetic nanoparticles. The method achieved high sensitivity and specificity, identifying single-base mismatches and achieving femtomolar detection limits, demonstrating its potential for advanced diagnostics (293).

Surface of exosomes can also be identified for active targeting, increasing the circulation time and producing the specific drug target vehicle (294) For instance, Stickney Z et al. developed a surface display technology using exosomes and tetraspanins to address the lack of a suitable mammalian display system. They created fluorescent reporters for both inner and outer display, demonstrated successful display, and validated the system *in vivo*. Their work has potential applications in exosome tracking, targeted drug delivery, and vaccines (295). Additionally, the use of aptamer-drug-exosome has been shown to have higher affinity to tumor environment other than drug-exosome alone (294). Zuo et al. created an aptamer-equipped exosomes (Exos) platform for effective chemotherauetic delivery to cancer targets using diacyllipid–aptamer conjugates. The aptamer-modified Exos (Apt-Exos) can specifically deliver drugs to cancer cells, providing an efficient delivery platform for targeted cancer therapy and diagnosis (296).

Because exosome profiling will contain massive data, machine learning and bioinformatics play an essential role in improving the analytical tools (288). The study developed a prognostic model using disulfidptosis-related gene expression patterns for breast cancer. It identified key DRGs and used unsupervised clustering to define immune subtypes. The model validated four DRGs, indicating potential for personalized treatment strategies (297).

Several ongoing clinical trials are exploring the diagnostic and therapeutic potential of EVs in various cancers (**Table 4**). These trials focus on utilizing EVs as non-invasive tools for early cancer detection, detection of various cancer-related mutations, and disease monitoring, offering a promising alternative to traditional invasive methods. Trials are furthermore assessing the role of EVs in predicting treatment responses, particularly in immunotherapy and targeted therapies by analyzing several EV-based biomarkers (298). Additionally, EVs are being explored as potential therapeutic agents, including their use in cancer vaccine development and drug delivery systems (298). These advancements highlight the growing interest in EV-based applications for diagnosing and treating cancer, however further research is necessary to fully understand their clinical benefits and practical applications.

**Table 4:** Current clinical trials on clinical applications of extracellular vesicles in cancer.

Cancer type	Cancer subtype	Clinical use	Description	Trial status	NCT identifier
Lung Cancer	Adenocarcinoma	Mutation Detection	EGFR mutation identification using EV-based BALF liquid biopsy	Recruiting	NCT05469022
		Treatment Response	Detection of plasma exosomes to predict response of EGFR mutation targeted therapy	Enrolling by invitation	NCT06730477
		Diagnostic Biomarker	Detection of exosomes through deep-learning analysis to diagnose NSCLC	Unknown	NCT04529915
	NSCLC	Tumor Vaccination	Tumor vaccination with tumor antigen-loaded dendritic cell-derived exosomes in unresectable NSCLC	Completed	NCT01159288
		Mutation Detection	Detection of EML4-ALK fusion by plasma exosomes	Recruiting	NCT04499794
		Treatment Response	Detection of small-EV miRNAs as biomarkers for Anlotinib efficacy in NSCLC	Not yet recruiting	NCT05218759
		Treatment Response	Detection of exosomal PD-L1 and miRNA expression as predictors of immunotherapy response	Unknown	NCT04427475
		Treatment Response	Detection of exosomal PD-L1 mRNA expression as predictors of radioimmunotherapy response	Completed	NCT02869685
		Disease Monitoring	EVs as biomarkers to detect cancer recurrence	Recruiting	NCT05424029
		Disease Monitoring	Detection of CNS metastasis using exosomes as predictors of lung cancer metastasis	Recruiting	NCT06026735
		Diagnostic Biomarker	Detection of EVs via liquid biopsy for the diagnosis of lung cancer	Recruiting	NCT05587114
		Disease Monitoring	Molecular profiling of exosomes as potential biomarkers for cancer recurrence	Active	NCT04939324
	SCLC	Treatment Response	Detection of circulating EV Long RNA profile for predicting SCLC treatment response	Unknown	NCT05191849
	Squamous Cell	Treatment Response	Serum exosomal miRNA combined with PD-L1 as biomarkers to predict anti-PD-L1 immunotherapy response	Recruiting	NCT05854030
	Unspecified	Diagnostic Biomarker	Exosomal assays based on hypoxia detection as biomarkers for early detection	Unknown	NCT04629079
		Diagnostic Biomarker	Detection of exosomal RNA for the diagnosis and identification of malignant lung nodules	Unknown	NCT04182893
		Diagnostic Biomarker	Detection of exosomal RNA using RNA sequencing as a biomarker for early diagnosis	Completed	NCT03830619
Brain Cancer	Meningioma	Treatment Response	Detection of plasma EVs during and post meningioma radiotherapy	Recruiting	NCT06104930
	Retinoblastoma	Mutation Detection	Detection of RB-1 mutation tumors using EV-based tests	Completed	NCT04164134
Breast Cancer		Diagnostic Biomarker	Detection of glycosylated EVs containing miRNAs for early diagnosis	Recruiting	NCT05417048
		Diagnostic Biomarker	Detection of tumor-derived-EVs associated proteins as biomarkers	Recruiting	NCT05798338
		Risk Stratification	Stratification of breast cancer risk by the detection of EVs in blood using a liquid biopsy spectroscopy-based device	Recruiting	NCT06672302
		Diagnostic Biomarker	Detection of exosomes as prognostic and predictive biomarkers	Active	NCT05955521
		Disease Monitoring	Detection of meningeal metastasis in breast cancer by exploring association between proteomic profiles of CSF microvesicles and CSF cytology	Completed	NCT05286684
		Therapeutic Response	Detection of tumor-derived-EVs associated proteins to assess efficacy of neoadjuvant treatment	Recruiting	NCT05831397
Genitourinary Cancer	Bladder	Disease Monitoring	Detection of exosome IncRNA-ELNAT1 as a predictor of lymph node metastasis	Not yet recruiting	NCT05270174
	Prostate	Treatment Response	Detection of tumor EVs response to radical prostatectomy	Recruiting	NCT06326216
		Diagnostic Biomarker	Detection of plasma exosome RNA for diagnosis	Enrolling by invitation	NCT06604130
	Ovarian	Diagnostic Biomarker	Exosome-based prediction model for ovarian cancer prediction	Not yet recruiting	NCT06558019
		Diagnostic Biomarker	Exosomal miRNA and IncRNA detection for the diagnosis of ovarian cancer	Unknown	NCT03738319
	Renal	Diagnostic Biomarker	Detecting tumor-based exosomes for the early diagnosis of clear cell renal cell carcioma	Recruiting	NCT04053855
		Treatment Response	Detecting exosomes to predict immunotherapy response in metastatic renal cell carcinoma	Recruiting	NCT05705583
Gastrointestinal Cancer	Pancreatic	Diagnostic Biomarker	EV-bound protein biomarkers used in an assay for detection of high-risk pancreatic adenocarcinoma	Recruiting	NCT05625529
		Diagnostic Biomarker	Exosomes as biomarkers for pancreatic cancer diagnosis, disease recurrence, and outcomes	Recruiting	NCT02393703
		Diagnostic Biomarker	Exosomes as biomarkers for pancreatic cancer diagnosis	Recruiting	NCT03334708
		Diagnostic Biomarker	Exosome detection by liquid-based biopsy for diagnosis	Enrolling by invitation	NCT06108531
		Diagnostic Biomarker	Detection of exosomal miRNA for diagnosis and early detection	Recruiting	NCT06388967
		Diagnostic Biomarker	Detection of exosomal small RNAs for the diagnosis of pancreatic cancer	Unknown	NCT04636788
		Vehicle	Mesenchymal stromal cells-derived exosomes with KrasG12D siRNA as cargos	Active	NCT03608631
	Esophageal & Gastric	Diagnostic Biomarker	Proteomic analysis of plasma exosomes for the early detection of upper gastrointestinal cancers	Recruiting	NCT06278064
	Gastric	Treatment Response	EV-based score to predict immunotherapeutic outcomes of gastric cancer	Unknown	NCT04993378
		Diagnostic Biomarker	Detecting exosomal IncRNA-GC1 as diagnostic biomarkers	Unknown	NCT05397548
		Diagnostic Biomarker	Detecting tumor-based exosomes as prognostic and predictive biomarkers	Unknown	NCT01779583
		Diagnostic Biomarker	Detecting exosomal microRNA and cell-free microRNA for early diagnosis of gastric cancer	Completed	NCT06342427
	Colorectal	Treatment Response	Detection of tumor EVs response to neoadjuvant chemoradiotherapy in rectal cancer	Recruiting	NCT04852653
		Treatment Response	Detection of tumor exosomal response to neoadjuvant chemoradiotherapy	Recruiting	NCT03874559
		Treatment Response	Detection of tumor exosomal as predictive models to monitor response to neoadjuvant chemoradiotherapy	Unknown	NCT04227886
		Diagnostic Biomarker	Detection of exosomal-derived miRNA combined with cell-free miRNA as biomarkers for early diagnosis	Recruiting	NCT06342440
		Disease Monitoring	Tumor-derived exosomes containing miRNAs as biomarkers of early prognosis in colon cancer	Unknown	NCT04523389
		Diagnostic Biomarker	Detection and characterization of exosomes	Completed	NCT04394572
		Vehicle	Curcumin delivery via exosomes	Recruiting	NCT01294072
		Diagnostic Biomarker	Detection of cell-free and exosomal miRNAs via liquid biopsy as biomarkers for early onset cancer	Recruiting	NCT06342401
		Disease Monitoring	Exosome-based liquid biopsy assay using exosomal miRNAs to detect molecular residual disease in colon cancer	Recruiting	NCT06654622
	Gallbladder	Diagnostic Biomarker	Detection and correlation of exosomes to gallbladder carcinoma by proteomic studies	Unknown	NCT03581435
	Hepatocellular Carcinoma	Treatment Response	Detection of exosomal PD-L1 and LAG-3 proteins to assess immunotherapy response	Unknown	NCT05575622
		Diagnostic Biomarker	Detection of exosomal miRNAs via liquid biopsy to aid in the diagnosis between hepatocellular carcinoma and intrahepatic cholangiocarcinoma	Recruiting	NCT06342414
	Cholangiocarcinoma	Diagnostic Biomarker	Characterization of ncRNAs of tumor-derived exosomes	Unknown	NCT03102268
		Disease Monitoring	Detection of exosomal miRNAs via liquid biopsy as predictors for lymph node metastasis	Recruiting	NCT06381648
Thyroid Cancer	Follicular Thyroid Cancer	Diagnostic Biomarker	Detection of urinary exosomal cargos including thyroglobulin, calprotectin A8/A9, and Annexin-2, as biomarkers for diagnosis	Recruiting	NCT05463107
	Unspecified	Disease Monitoring	Detection of exosomal thyroglobulin and galectin-3 for the prediction of cancer prognosis and recurrence	Completed	NCT03488134
		Diagnostic Biomarker	Detection of thyroid-derived EVs for thyroid cancer prediction	Suspended	NCT04742608
		Diagnostic Biomarker	Detection of exosomal thyroglobulin and galectin-3	Recruiting	NCT04948437
Oral Cancer	Unspecified	Disease Monitoring	Salivary miRNA containing-EVs as biomarkers for malignant transformation	Completed	NCT04913545
	Oropharyngeal Squamous Cell Carcinoma	Screening	Detection of exosomal HPV proteins to screen HPV-positive oropharyngeal cancer	Recruiting	NCT02147418
		Disease Monitoring	Detection of exosomal miR-185 to assess malignant transformation of oral leukoplakia	Completed	NCT06469892
Bone and Soft Tissue Cancer	Osteosarcoma	Disease Monitoring	Exosomes containing RNA cargos to detect lung metastases in primary osteosarcoma	Active	NCT03108677
		Disease Monitoring	Detection of exosomes as biomarkers by microfluidic chip technology for early diagnosis of osteosarcoma lung metastasis	Completed	NCT05101655
	Sarcoma	Disease Monitoring	Detection of sarcoma-derived exosomes for disease monitoring	Recruiting	NCT03800121
Skin Cancer	Melanoma	Disease Monitoring	Detection of disease progression via PD-L1 labeling in exosomes as biomarkers	Active	NCT05744076
		Pathogenesis	Role of exosomes in melanoma development. Progress, and drug resistance	Completed	NCT02310451

The clinical translation of EVs as diagnostic and therapeutic modalities in cancer treatment is currently limited due to several significant challenges. Key challenges include the large-scale isolation of EVs, which remains a complex and costly process, and the heterogeneity of EV populations, which complexes standardization (288). Additionally, scalability and reproducibility in biomarker detection are concerns, as consistent isolation and characterization techniques are crucial for reliable results. The incremental validation of EV-based therapies presents another hurdle, requiring extensive preclinical and clinical testing to establish their safety and efficacy (288). Furthermore, navigating the regulatory landscape for EV-based therapeutics poses substantial challenges, given the need for rigorous validation and approval processes. Overcoming these limitations is essential for the successful clinical application of EVs in cancer therapy.

## 11 Conclusion

The field of cancer diagnostics and therapeutics is transforming through extra-cellular vesicles, as they present a minimally invasive precise platform for disease management. Through their biomarker role EVs enable important understanding of tumor evolution as well as therapy-related adjustments thereby enabling medical staff to detect tumors early and deliver personalized care. The carriers transport drugs effectively with decreased side effects in the body. Several obstacles stand in the way of clinical deployment because standard isolation methods need improvement alongside scale-up methods and the development of better detection accuracy. The enhancement of clinical diagnostics through EV-based methods can be achieved by implementing advanced EV-based techniques. Future innovations and translational work in the field must continue because ongoing research studies show EVs have a major role in changing cancer treatment through their clinical significance.
